# Therapies and Vaccines Based on Nanoparticles for the Treatment of Systemic Fungal Infections

**DOI:** 10.3389/fcimb.2020.00463

**Published:** 2020-09-03

**Authors:** Brenda Kischkel, Suélen A. Rossi, Samuel R. Santos, Joshua D. Nosanchuk, Luiz R. Travassos, Carlos P. Taborda

**Affiliations:** ^1^Department of Microbiology, Institute of Biomedical Sciences, University of São Paulo, São Paulo, Brazil; ^2^Laboratory of Medical Mycology-Institute of Tropical Medicine of São Paulo/LIM53/Medical School, University of São Paulo, São Paulo, Brazil; ^3^Departments of Medicine [Division of Infectious Diseases], Microbiology and Immunology, Albert Einstein College of Medicine and Montefiore Medical Center, Bronx, NY, United States; ^4^Department of Microbiology, Immunology and Parasitology, Federal University of São Paulo, São Paulo, Brazil

**Keywords:** drug delivery systems, vaccine adjuvant, antifungal therapy, mycosis, *Candida albicans*, *Cryptococcus* sp., *Histoplasma capsulatum*

## Abstract

Treatment modalities for systemic mycoses are still limited. Currently, the main antifungal therapeutics include polyenes, azoles, and echinocandins. However, even in the setting of appropriate administration of antifungals, mortality rates remain unacceptably high. Moreover, antifungal therapy is expensive, treatment periods can range from weeks to years, and toxicity is also a serious concern. In recent years, the increased number of immunocompromised individuals has contributed to the high global incidence of systemic fungal infections. Given the high morbidity and mortality rates, the complexity of treatment strategies, drug toxicity, and the worldwide burden of disease, there is a need for new and efficient therapeutic means to combat invasive mycoses. One promising avenue that is actively being pursued is nanotechnology, to develop new antifungal therapies and efficient vaccines, since it allows for a targeted delivery of drugs and antigens, which can reduce toxicity and treatment costs. The goal of this review is to discuss studies using nanoparticles to develop new therapeutic options, including vaccination methods, to combat systemic mycoses caused by *Candida* sp., *Cryptococcus* sp., *Paracoccidioides* sp., *Histoplasma* sp., *Coccidioides* sp., and *Aspergillus* sp., in addition to providing important information on the use of different types of nanoparticles, nanocarriers and their corresponding mechanisms of action.

## Introduction

Fungal diseases are broadly classified according to the degree of interactions between the pathogen and the host tissue in superficial, subcutaneous, and systemic infections (Tiew et al., 2020). Superficial mycoses, which are estimated to occur in 25% of the world population, are the most common form of fungal infection. Systemic mycosis, however, is most severe since it is associated to a high mortality rate, significant morbidity, limited chemotherapeutic options, and the diagnosis is frequently difficult and complex (Kauffman, 2007; Brunet et al., 2018).

Opportunistic fungal infections usually occur in immunocompromised individuals as a result of subjacent infection or the treatment itself. Infections of endogenous origin caused by pathogens such as *Candida albicans* can also occur (Colombo et al., 2017; Rautemaa-Richardson and Richardson, 2017). Species of *Aspergillus, Candida, Cryptococcus*, and *Trichosporon* are the main agents of opportunistic mycoses. Endemic fungal infections are usually caused by dimorphic fungi, found in the soil or in animal feces. Host acquisition occurs by inhalation of infectious spores/infective propagules (Rodríguez-Cerdeira et al., 2014). In the case of endemic mycoses, immunocompetent individuals dwelling in endemic areas may develop severe disease following inhalation of fungal particles, associated or not to a competent immune response (Edwards et al., 2013). The main species that cause endemic mycoses are *Blastomyces dermatitidis, Coccidioides immitis* and *Coccidioides posadasii, Histoplasma capsulatum, Paracoccidioides*, particularly *P. brasiliensis* and *P. lutzii, Sporothrix*, primarily *S. brasiliensis* and *S. schenckii*, and *Talaromyces marneffei* (Brown et al., 2012; Limper et al., 2017).

In the second half of the 20th century, a worldwide, progressive increase in the number of immunocompromised individuals took place, paralleling the outcome of HIV epidemics as well as the expanded use of immunosuppressive drugs in cancer, autoimmune disease, and transplant patients (Coelho and Casadevall, 2016; Armstrong-James et al., 2017). As a consequence, systemic mycoses have been considered an emergent threat, because immunocompromised individuals are more susceptible to fungal infection (Lockhart, 2019). Cryptococcosis is an excellent example of the profound impact of fungal infections over time, in humans. The number of infections caused by *Cryptococcus* has increased from 300 cases in 1,950 to ~1 million cases in 2008 causing ~600,000 deaths per year in patients with HIV (Park et al., 2009; Del Poeta and Casadevall, 2012). Currently this number is closer to ~180,000 deaths annually (Rajasingham et al., 2017). Globally, the estimated number of deaths per year has been 6 million among invasive fungal infections (Stop neglecting fungi, 2017). In the case of invasive *Candida* and *Aspergillus* infections, the mortality rate could reach 60–80%, respectively (Perlroth et al., 2007; Moriyama et al., 2014). Bongomin et al. (2017) estimated the number of mycoses in the Leading International Fungal Education (LIFE) portal, which covers 80% of the world's population (5.7 billion people), and estimated that there occurred annually ~3,000,000 cases of pulmonary aspergillosis, ~250,000 of invasive aspergillosis, ~700,000 of invasive candidiasis, and ~500,000 of histoplasmosis of which ~100,000 were disseminated cases. The global estimate of *Paracoccidioides* and *Coccidioides* cases is 4,000 and 25,000, respectively. It must be emphasized that these are considered neglected diseases, therefore the number of reported cases can be an underestimate of the actual burden of the disease. In Brazil, paracoccidioidomycosis (PCM) ranks as the 8th death causing infectious disease in patients without immunosuppression, surpassing histoplasmosis, or cryptococcosis in this group of patients. In fact, there is no compulsory notification of fungal infections in Brazil and the disease frequently occurs in rural and poor farmer populations. They frequently lack access to medical care, therefore the presumed incidence of the mycoses that differ from the real one (Shikanai-Yasuda and Mendes, 2007; Giacomazzi et al., 2016).

Fungal infections are often defined as difficult to treat, including the toxicity of antifungals and their interaction with other drugs (Westerberg and Voyack, 2013; Bicanic, 2014; Brunet et al., 2018). There is broad consensus that currently available antifungal therapy is limited and far from ideal (LaSenna and Tosti, 2015; Armstrong-James et al., 2017; Brunet et al., 2018). In the USA, fungal diseases may cost more than 7.0 billion dollars a year (Benedict et al., 2019), and the treatment of invasive fungal infections 70,000 dollars per patient (Ashley et al., 2012). Particularly in under resourced populations, the long periods and high costs of treatment contribute to patients abandoning their chemotherapy, when clinical symptoms may disappear, but are frequently followed by disease recurrences.

Systemic fungal infections are largely treated with polyenes, azoles or echinocandins, depending on the fungal pathogen and the clinical condition of the patient (Polvi et al., 2015; Souza and Amaral, 2017). The traditional antifungal agent is amphotericin B (AmB), a polyene with a broad spectrum of action, involving interaction with fungal ergosterol, destabilization of the cell membrane and, consequently, the death of the pathogen (Palacios et al., 2011). The drug interacts also with mammalian sterols, such as cholesterol, which can lead to treated patient collateral effects (Carmona and Limper, 2017). Azoles are widely prescribed against invasive fungal infections, mainly represented by fluconazole, voriconazole, itraconazole (ITZ), posaconazole, and isavuconazole (Gintjee et al., 2020). Azoles act by inhibiting lanosterol 14α-demethylase (Erg11), which converts lanosterol into ergosterol (Di Mambro et al., 2019). Its activity is also associated to inhibition of cytochrome P450 with undesired side effects. Echinocandins, micafungin, caspofungin, and anidulafungin target, on the other hand, receptors that do not exist in human cells such as β(1,3)-D-glucan synthase, an enzyme responsible for the synthesis of β-1,3 glucan, a structural component of the fungal cell wall (Di Mambro et al., 2019). Such reactivity makes echinocandins more tolerable, with limited toxicity and drug interaction. However, a limited spectrum of action is exhibited toward certain yeasts and molds, with no activity against important opportunistic yeasts such as *Cryptococcus* sp. and dimorphic fungi (Lewis, 2011; Gintjee et al., 2020).

Azole resistance is well-recognized in *Aspergillus fumigatus, Cryptococcus neoformans, Coccidioides* spp., *H. capsulatum*, and *Candida* sp. (Wheat et al., 2001; Kriesel et al., 2008; Snelders et al., 2011; Vincent et al., 2013; Fontes et al., 2017). The resistance to azoles is mainly due to mutations in fungal DNA, which reduce the interactions between the drug and the cell target (Hagiwara et al., 2016). As examples, in *A. fumigatus* azole resistance mechanisms include the insertion of repeated sequences in tandem into the *cyp51A* promoter, amino acid substitutions in the structure of the target Cyp51A protein, and overexpression of the ABC transporter Cdr1B (Hagiwara et al., 2016). Although rare, resistance to AmB can occur intrinsically or it may be induced. *Candida tropicalis* resists the action of AmB by reducing mitochondrial production of reactive oxygen species (ROS) (Vincent et al., 2013). In *Aspergillus terreus* the genes encoding catalase (CAT) and superoxide dismutase (SOD) are essential for intrinsic resistance, since the inhibition of these enzymes makes the isolates susceptible to treatment by the drug (Jukic et al., 2017). Low levels of β-1,3 glucan lead to the lack of efficacy of echinocandins against certain species, but resistance can also develop, primarily through hotspot mutations, such as changes in glucan synthase genes (Huang et al., 2016).

An intact immune system prevents the development of most invasive fungal infections. Hence, there is significant interest in stimulating the immune system to get a more effective response against pathogenic fungi primary or during treatment of the installed disease. Studies supported that therapeutic or prophylactic vaccines can stimulate the immune system in experimental mycosis models, even in immunosuppressed mice (Silva et al., 2017). The combination of vaccination and antifungal chemotherapy leads to improved treatment efficacy and reduction of treatment period, which would also potentially prevent relapses (Travassos and Taborda, 2017). Currently there is no licensed vaccine, prophylactic or therapeutic, to treat human systemic mycoses (Travassos and Taborda, 2017). Experimental vaccines have been developed for histoplasmosis, aspergillosis, candidiasis, cryptococcosis, coccidioidomycosis, and PCM (Brown et al., 2012), but none have progressed to market. Such delay is linked to a myriad of obstacles, which include lack of adequate formulation, high development costs, and lack of market interest (Cassone and Casadevall, 2012). In addition, the fact that some fungal diseases mainly affect immunocompromised individuals is an obstacle to the generation of an effective vaccine for this population. Currently, different research groups have focused on the development of a vaccine that can be used both in healthy patients and in immunodeficient ones or otherwise high-risk patients, providing protection without aggravating the patient's clinical condition (Spellberg, 2011; Cassone and Casadevall, 2012; Medici et al., 2015; Travassos and Taborda, 2017). Additionally, the identification of appropriate adjuvants has been a major obstacle for fungal vaccine development.

Nanotechnology is a field that has been widely explored as an innovative and low-cost strategy for the development of new antifungals and more efficient vaccines (Souza and Amaral, 2017). This application of nanotechnology in vaccine development has attracted the attention of researchers since nanotherapeutics can utilize low toxicity materials that allow for the slow and direct delivery of drugs and antigens to specific targets (Zhao et al., 2014). In relation to antifungal chemotherapy, nanoparticles (NPs) have been used due to their intrinsic antifungal activity or as a drug delivery vehicle with a focus on reducing the concentration of drug required for treatment (Zhao et al., 2014). In the formulation of vaccines, NPs can act as a delivery tool capable of improving the stability of antigens such as peptides and the immunogenicity of the antigen, as well as possible immunostimulant adjuvants (Zhao et al., 2014).

In this review, we discuss the use of NPs in the development of new therapeutic approaches and vaccines against systemic mycoses, briefly commenting on the types of NPs used for this purpose and their mechanism of action. Finally, we present the current state of art of NPs for the development of new antifungal agents and vaccines aiming at systemic mycoses with a focus on *Candida* sp., *Cryptococcus* sp., *Paracoccidioides* sp., *Histoplasma* sp., *Coccidioides* sp., and *Aspergillus* sp..

## NPs and the Development of a New Therapeutic Approach and Vaccination Alternative

The treatment of systemic fungal infections has limitations since currently available antifungals exhibit low biodistribution and treatment effectiveness, with lack of selectivity, and serious side effects (Voltan et al., 2016). Nanotechnology appears as an alternative to these problems since NPs can function as a controlled and specific drug delivery system, which can improve mycosis treatment without impairing the patient's quality of life.

NPs can be obtained by physical, chemical, or biological methods. The synthetic process should consider constraints of large-scale production, stability, cost, and toxicity. Methodologies involving physical synthesis can be expensive, particularly due to the equipment used for electronic excitation (Haroon Anwar, 2018). Inorganic solvents used in the chemical reduction are highly toxic, including citrate, borohydride, thioglycerol, and 2-mercaptoethanol (Zhang X.-F. et al., 2016). The biological synthesis of NPs can significantly reduce the risk of producing toxic compounds by employing plant extracts, or bacterial and fungal metabolites with antimicrobial potential, acting as reducing agents, and/or stabilizers of NPs (Ahmed et al., 2018; Lakshmeesha et al., 2019). In addition to developing NPs appropriate for medical application, the nanoformulation is also essential since the efficacy of biological activity and cytotoxicity depends on the physicochemical properties exhibited by NPs, such as size, shape, surface area, solubility, aggregation, composition with coating reactivity of particles in solution, ion release efficiency, and type of the reducing agent used in the synthetic process (Carlson et al., 2008; Murdock et al., 2008; Lin et al., 2014).

NPs exhibit antimicrobial activity through different mechanisms. Nanometric particles can cross the cell interstitium and release metal ions from the surface of the NPs inside the cell, increasing the antimicrobial activity, due to their interaction with proteins, inhibiting their activity or causing damage to the cell wall, leading to pathogen death (Oberdörster et al., 2005; Reddy et al., 2012). Another mechanism of action is through oxidative stress, which can vary based on the specific chemical properties of the materials, such as the formation of surface groups that act as reactive sites. Active sites reacting with O_2_ lead to the formation of ROS that increase tissue damage (Nel et al., 2006). Oxidation of fatty acid double bonds in cell membranes, may alter membrane permeability and increase the osmotic stress resulting in cell death. In addition, ROS can damage the DNA, RNA, and proteins of the pathogen (Reddy et al., 2012; Huang et al., 2014; Rónavári et al., 2018; Rodrigues et al., 2019). Increased toxicity is inversely proportional to NPs size. Specifically, small NPs have a larger gravimetric specific surface area, which allows more molecules to be exposed for interaction and raising damage (Nel et al., 2006).

The antimicrobial activity of NPs against pathogens *in vitro* and *in vivo* has been extensively reported in the literature (Ambrosio et al., 2019; Lakshmeesha et al., 2019; Xue et al., 2019). Nanoformulations showing a broad spectrum of action by inhibiting the growth of different pathogens such as fungi, bacteria or viruses, such as silver NPs (AgNPs), have been described (Yah and Simate, 2015). Mohammed Fayaz et al. (2012) developed a method for coating polyurethane condoms with Ag and demonstrated that the product was able to inactivate HIV-1/2 and significantly inhibit the growth of bacteria (*Escherichia coli, Staphylococcus aureus, Micrococcus luteus*, and *Klebsiella pneumoniae)* and *Candida* (*C. tropicalis, C. krusei, C. glabrata*, and *C. albicans*). In addition, chitosan-carbon nanotube (Chitosan-CNT) hydrogels, exploited in medicine for dressing and drug administration applications, inhibited the growth of *S. aureus, E. coli*, and *C. tropicalis* (Venkatesan et al., 2014).

The ability to form a biofilm is an important virulence mechanism that microorganisms such as bacteria and fungi are able to build during infection, which is also associated with disease persistence as well as relapses (Wojtyczka et al., 2013; Sav et al., 2018). The antibiofilm activity of NPs has been studied and the potential of nanoformulations to disrupt these complex matrices have been reported (Khan et al., 2012; Gondim et al., 2018; Yang et al., 2019).

Nanotechnology effectively combat both extracellular and intracellular pathogens. In the case of extracellular pathogens, biocompatibility and the time spent in the bloodstream in adequate concentrations is essential for the successful treatment of systemic infections. Therapy for intracellular pathogens requires a more nuanced approach that includes the proper targeting of nanocarriers to infected cells through different ligands. In general, drugs targeting intracellular pathogens have to disrupt or transit the cell membrane and release and maintain the drug at the therapeutic level for the desired time inside the target cell. Certain nanocarriers enter the cell through endocytic mechanisms and remain stable within the endolysosomes until they gain access to the cytosol to release the drug. Otherwise, the release of the drug into the endolysosome could render it ineffective (Armstead and Li, 2011). The nanometric scale of particles allows for drug delivery into specific locations of the body, entering living cells to deliver drug or antigen payloads into macrophages and dendritic cells, which is particularly useful in the treatment of intracellular pathogens such as *H. capsulatum* (Couvreur, 2013; Dube et al., 2014). Dendritic cells are capable of absorbing particles of 20–200 nm, whereas particles of 0.5–5 μm are taken up by macrophages (Xiang et al., 2006). Receptors on these cells act, thus, to improve antigen processing and activate pathways that will enhance the immune response (Zhao et al., 2014). An example is β-1,3-glucan, a polysaccharide found in the cell wall of most fungi that interacts directly with the Dectin-1 receptor present on the surface of macrophages (Brown et al., 2002). Activation of Dectin-1 enhances phagocytosis and consequently promote a greater absorption of NPs into macrophages (Goodridge et al., 2009). Several studies have validated that nanoparticles can target intracellular pathogens; however, most of these studies have targeted the bacterium *Mycobacterium tuberculosis* or the parasite *Leishmania brasiliensis* (Dube et al., 2014; Tukulula et al., 2015, 2018). Poly (lactic-co-glycolic acid) (PLGA) NPs made functional by β-1,3-glucan and carrying rifampin have shown promise for the treatment of tuberculosis, and these particles were not cytotoxic and were quickly recognized by macrophages (Tukulula et al., 2015, 2018). Chitosan—PLGA core-shell NPs with β-1,3 glucan and rifampin, increased the intracellular concentration of rifampin and showed an enhanced ability to modulate immune responses of human alveolar macrophages (Dube et al., 2014). In addition to β-glucan, other types of ligands such as antibodies can be incorporated on the surface of nanocarriers to target specific compartments of the target cell to act against intracellular pathogens. The use of pH-responsive polymers, which specifically release drugs in the presence of defined pHs, also represents an attractive alternative that can be explored against fungal pathogens (Armstead and Li, 2011). Interestingly, Mehta et al. (1997) suggested that the liposomal AmB synthesized by the authors in the 7:3 ratio of DMPC: DMPG (similar to Abelcet) is captured and retained by macrophages. These macrophages demonstrated enhanced killing of yeast cells, in this case *C. albicans*. However, this was not due to differential activation of the macrophages. The authors proposed that the candidicidal activity of the formulation occurred due to the macrophages retaining the liposomal AmB and releasing the drug to kill the yeast (i.e., drug delivery via macrophages).

**Figure 1:**
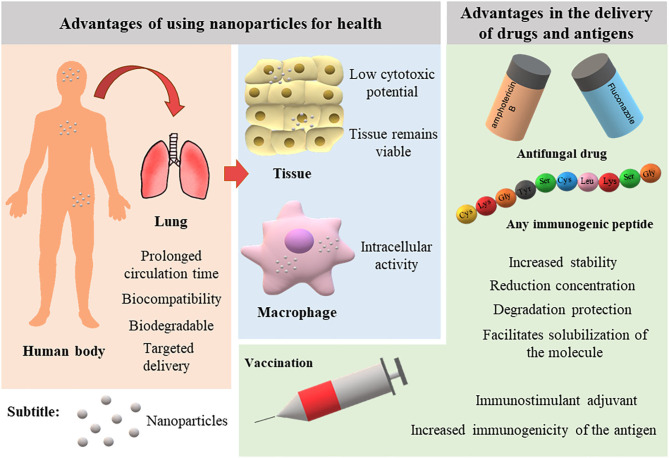
Advantages of nanoparticles for therapy and vaccination of infectious diseases.

Nanotechnology has advanced in recent decades with the development of innovative nanoscale products for various applications. Figure 1 illustrates the main advantages of using NPs in the medical field. Currently, the nanomedicine market includes new approaches in the diagnosis, prevention, and therapy of diverse diseases. In 2006, these innovations sustained a market of US $6.8 billion (Wagner et al., 2006). A recent study predicted an annual growth of 12.6% such that the market can reach up to US $261 billion in 2023 (reviewed by Marques et al., 2019).

## Types of Nanocarriers

Several types of nanostructures are currently being investigated for the delivery of antifungal drugs and improve their ability to serve as adjuvants for vaccine delivery (Ribeiro et al., 2013; Souza and Amaral, 2017). These nanostructures can be classified according to their composition into (Soliman, 2017): polymeric NPs, phospholipid-based vesicles, nanostructured lipid carriers (NLCs), dendrimers, nano-emulsions (NE), and metallic and magnetic NPs. Below, we briefly present the types of nanocarriers that can be used in formulations for systemic administration of antifungals and antigen delivery.

### Polymeric Nanoparticles

The most common materials used for nano-carrier development are polymers (Bolhassani et al., 2014). Polymeric NPs are formed by chains of identical chemical structures, called monomers. Polymers are generated by the union of several monomers (Sahoo et al., 2007). Polymers and monomers may be extracted from nature or chemically synthesized. The main polymers used in the production of NPs for mycosis treatment are alginate, chitosan, and PLGA (Italia et al., 2011; Yang et al., 2011; Spadari et al., 2017; Fernandes Costa et al., 2019).

Alginate is a natural polymer found mainly in the cell wall of algae of the Phylum *Phaeophyta*, and it is formed by the junction of two monomers, α-L-guluronic acid (G block), and β-D-mannuronic acid (M block) (Jain and Bar-Shalom, 2014). The difference in concentrations between the monomers and the variations in their arrangement defines how rigid the polymer structure will be, and consequently the NPs (Jain and Bar-Shalom, 2014). Alginate NPs can be obtained through different techniques that work in basically the same way, with alginate interacting with calcium salts and promoting polymer folding to form NPs (Jain and Bar-Shalom, 2014; Lopes et al., 2017). Alginate is a water-soluble polymer, and alginate-based NPs have biocompatible mucoadhesive characteristics and are non-cytotoxic (Yehia et al., 2009).

Chitosan is a polymer obtained from the deacetylation of chitin, which is widely distributed in nature, particularly in the animal and fungal kingdoms. In the animal kingdom, chitin is present in insect, arachnid, and crustacean exoskeletons, whereas fungal chitin is a cell wall component in most fungi (Frank et al., 2020). Chitosan may be used for the production of nanogels, nano-emulsions, and NPs. Among the different applications of chitosan are food supplementation, wound healing, and immunomodulation (Dai et al., 2011; Ahmed and Aljaeid, 2016). Due to its hydrophobic character and positive charge, chitosan is ideal for the production of NPs and delivery of different types of molecules in mucous membranes (Frank et al., 2020). DNA/RNA, peptides, proteins, and drugs (Illum, 2003; Riteau and Sher, 2016) are effectively delivered by chitosan NPs. Since chitosan is biocompatible, biodegradable, and non-cytotoxic, it is one of the most promising polymers for the development of vaccines or parenteral treatment in different types of systemic infections (Sharma et al., 2015; Frank et al., 2020).

PLGA is a synthetic co-polymer produced from the linkage of glycolic acid (GA) and lactic acid (LA) monomers, the same molecules produced by PLGA biodegradation (Amaral et al., 2009; Souza et al., 2015). The properties of PLGA are directly related to the molecular weight and proportions of the monomers. Therefore, the mechanical resistance, biodegradation rate, and the hydrolysis of the nanocarrier are influenced by the degree of crystallinity of the PLGA, which depends on the molar ratio between GA and LA. The most commonly used concentration being 50% poly lactic acid (PLA) and 50% poly glycolic acid (PGA) (Danhier et al., 2012). In addition, alkaline or strongly acidic pHs can accelerate the biodegradation of the polymer. Due to its biocompatibility, low cytotoxicity, and biodegradability, PLGA is one of the few Food and Drug Administration (FDA) approved polymers for use in complexing drugs or immunogenic molecules (Danhier et al., 2012). The production of PLGA NPs requires different techniques depending on the polarity of the molecules to be complexed (Amaral et al., 2010). PLGA NPs can be used for delivery of molecules via the enteral and parenteral routes, both followed by rapid body clearance (Semete et al., 2010). Pegylation, or the incorporation of polyethylene glycol (PEG) molecules on the NP surface, can make the NPs “invisible” to phagocytic cells and extend their half-life (Semete et al., 2010).

Amphiphilic block-copolymers, with hydrophilic shell and hydrophobic core, are used to form polymeric micelles, and these micelles can successfully deliver hydrophobic compounds. Polymeric micelles can improve drug administration and penetration, promoting drug accumulation in the target tissue. For these reasons, this type of nanocarrier has been explored to target drugs to the central nervous system, which is further discussed below in the topic on *Cryptoccocus* sp. (Shao et al., 2012).

### Phospholipid-Based Vesicles: Liposomes

Liposomes are lipid particles formed in a bilayer with a hydrophobic interior layer and a hydrophilic exterior, similar to the structure of a cellular plasma membrane (Nisini et al., 2018). Liposomes can be unilamellar (one bilayer) or multilamellar (several bilayers separated by some hydrophilic fluid), The feature of hydrophilic, hydrophobic, and hydrophilic spaces makes liposomes the most versatile particles for transporting molecules, which can be dispersed inside the lipid bilayer to interact with hydrophobic molecules or dispersed in the aqueous nucleus thus interacting with hydrophilic molecules. These features allow liposomes to carry large amounts of molecules and permits improved control over the release of these payload molecules (Lila and Ishida, 2017; Nisini et al., 2018).

Their similarity to plasma membranes provides another interesting facet of liposomes in that sterols can be added to modify the stiffness of the bilayer and liposomes can be used to anchor molecules that can direct and facilitate delivery of charged payloads. Liposomes can also be functionalized to simulate an infection; thereby an immune-like response can be stimulated reducing the need for adjuvants (Rukavina and Vanić, 2016; Kube et al., 2017; Lila and Ishida, 2017).

Several drugs have been incorporated into liposomes. Currently, formulations carrying AmB are commercially available as AmBisome® and Abelcet®. Ambisome® is a liposomal formulation of unicellular vesicles, formed from hydrogenated phosphatidylcholine from soy, cholesterol, distestylphosphatidylglycerol (DMPG) and AmB in the ratio 2:1:0.8:0.4. Abelcet® is a lipid complex with a multilamellar structure, formed of diesteroylphosphatidylcholine (DMPC) and DMPG in a 7:3 ratio, carrying 36 mol% of AmB. These formulations are adminstered worldwide to treat fungal infections (Newton et al., 2016; Godet et al., 2017).

### Nanostructured Lipid Carriers (NLCs)

NLCs are a mixture of solid lipid and a fraction of liquid lipid from natural sources, which make them biodegradable and biocompatible particles (Gartziandia et al., 2015; Khan et al., 2015). NLCs are second generation carriers that may overcome the disadvantages of solid lipid NPs (SLNs), which present low drug loading capacity and drug loss due to reorganization and formation of highly ordered crystalline arrangements during storage (Soliman, 2017). Thus, NLCs have improved characteristics due to the incorporation of a liquid lipid fraction that offers greater drug retention capacity and long-term stability, making this type of system more effective in drug delivery since most drugs are lipophilic in nature (Salvi and Pawar, 2019). ITZ incorporated into NLCs has shown more than 98% encapsulation efficiency in different studies and remained stable after 6-month storage (Pardeike et al., 2016; El-Sheridy et al., 2019). Beloqui et al. (2013) evaluated the tissue distribution of NLCs after intravenous administration in rats and confirmed that radiolabeled NLCs remain in circulation up to 24 h after administration. In addition, nanocarrier biodistribution is influenced by the particle size and charge. Large particles are captured by the lung and small particles by the liver and bone marrow, whereas positive NPs are observed in the kidney and negative NPs home to the liver. Therefore, NLCs have become valuable alternatives in drug delivery studies.

### Dendrimers

Dendrimers are highly branched polymeric NPs consisting of a multifunctional central core, branches, and end groups that allow functionalization (Sherje et al., 2018). Dendrimers can be constructed convergently (from edges to center) or divergently (from center to edges), and the form of construction is made in stages (generations) where each stage promotes uniform growth in size and shape because binding of branches is mirrored (Ahmed et al., 2016). Dendrimers are widely studied for the transport of drugs active against infections, inflammation, and cancer, or for the transport of genetic material such as DNA, RNA, or plasmids (Mendes et al., 2017). The main chemical components used for core construction are poly (amidoamine) (PAMAM), poly (propylene imine) (DAB or PPI), and poly (ether hydroxylamine) (PEHAM) (Voltan et al., 2016; Sherje et al., 2018).

Despite their wide range of applications, dendrimers may cause relevant cytotoxicity due to their composition, because the vast majority of dendrimers have a strong cationic characteristic that can cause membrane destabilization (Ghaffari et al., 2018; Sherje et al., 2018). However, various additions to these dendrimers have been introduced, which reduce the cytotoxic effects and prolong the body circulation time (Ghaffari et al., 2018).

### Nano-Emulsions (NE)

NE consist of isotropic mixtures of drugs, lipids, hydrophilic surfactants, and co-solvents, with droplet sizes ranging from 10 to 500 nm (Mundada et al., 2016). In general, they are kinetically stable and can replace less stable nanocarriers such as liposomes (Mahtab et al., 2016; Hussain et al., 2017). NE are of great interest as antifungal drug-delivery vehicles, since the lipophilic nature of the formulation permits the solubilization of drugs, which, coupled to the small size of the droplets, make them easily absorbed through biological membranes such as the intranasal mucosa (Thakkar et al., 2015; Hussain et al., 2016). Other authors have discussed intranasally administered NE as an efficient alternative for brain targeting drugs (Kumar et al., 2016; Chatterjee et al., 2019; Iqbal et al., 2019), including the analgesic Tramadol (Lalani et al., 2015) and the anti-depressive Paroxetine (Pandey et al., 2016). This approach is potentially relevant for the treatment of meningitis caused by *Cryptococcus* spp. Due to the versatility of NE in formulating gels, creams and foams, this approach has become widely explored in topical mycosis therapy (Jaiswal et al., 2015; Mahtab et al., 2016).

### Metallic and Magnetic Nanoparticles

Metallic NPs are extremely interesting, since, apart from acting as drug carriers, they represent an alternative to the treatment of infectious diseases via their intrinsic antimicrobial activity, which is well described for metals such as zinc, silver (Ag), and copper (Seil and Webster, 2012). Several studies have validated the intrinsic potential of metallic NPs in antimicrobial therapy (Franci et al., 2015; Malekkhaiat Häffner and Malmsten, 2017; Majid et al., 2018) and demonstrated their biocompatibility (Zhao et al., 2018). Silver is one of the noble metals most commonly used to generate NPs due to its unique properties such as chemical stability, good conductivity, and antimicrobial, antiviral, and antifungal potential as well as displaying anti-inflammatory activity (Ahmad et al., 2003). Metallic NPs have been widely explored in the literature for their synthesis from biological sources (Dipankar and Murugan, 2012; Thangamani and Bhuvaneshwari, 2019; Kischkel et al., 2020). The synthesis of NPs from plants or microorganisms is possible based on metabolites and proteins present in the extracts. These metabolites are essential for green synthetic pathways as they act to reduce metal and stabilize NPs (Khanna et al., 2019). Flavonoids, phenolic compounds, terpenoids, heterocyclic compounds, enzymes, and tannic acid are among the most commonly used compounds (Akhtar et al., 2013). Therefore, biologically synthesized NPs have the advantage of bringing together properties of the metal and the molecules used for synthesis (Dipankar and Murugan, 2012). Magnetic NPs can be formed from other metals such as iron, gold (Au), nickel, cobalt, and metal oxides (Huang et al., 2014). An advantage of using magnetic NPs is the ability to target their accumulation in the body, as magnetic NPs can be directed through a magnetic field generated by an external magnet to the specific site of drug delivery (Hussein-Al-Ali et al., 2014). This approach theoretically decreases the amount of drug needed for treatment and reduces drug concentration in non-target organs, which minimizes the incidence of serious side effects (Chomoucka et al., 2010; Rózalska et al., 2018; Rodrigues et al., 2019). In particular, superparamagnetic iron oxide NPs are a promising alternative for antifungal delivery, since they are highly responsive to external magnetic fields (Souza and Amaral, 2017).

**Figure 2:**
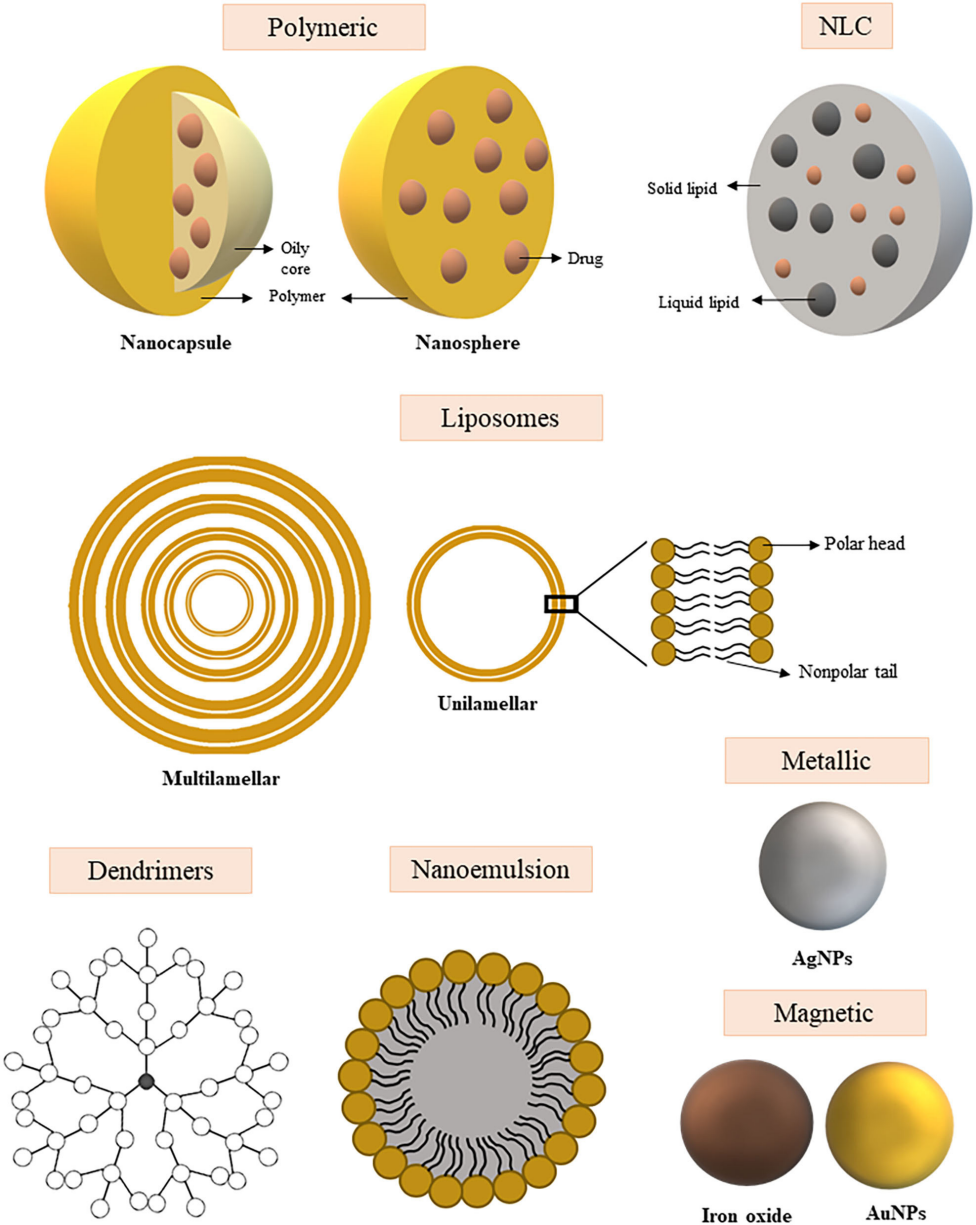
Schematic representation of the different types of nanoparticles used for the development of new therapeutic approaches and vaccination of systemic fungal infections. Polymeric nanoparticles: Nanocapsules contain an open core with drug space, surrounded by a polymeric membrane. The nanospheres carry the drugs evenly distributed over a polymeric matrix. NLC (Nanostructured lipid carriers): Structure composed of a solid lipid and liquid lipid fraction into which the drug may be incorporated within the structure. Liposomes: Nanoparticle with unilamellar or multilamellar structure with space for drug transport between layers and/or core. Dendrimers: Complex structure branched and highly organized around the nucleus, the drug can be incorporated between the layers or on the surface of the structure. Nanoemulsion: Colloidal dispersion composed of an oil and water phase that promotes drug encapsulation. Metallic nanoparticle: Nanoparticle of metal core and intrinsic antimicrobial activity. Magnetic nanoparticle: Nanoparticle of metal core with magnetic properties.

A schematic representation of the nanocarrier types described above can be seen in Figure 2.

## Current Scenario of Nanotechnology in the Treatment and Vaccination of Fungal Infections

### Candidiasis

Species of the genus *Candida* are part of the human microbiota. However, under conditions of immunosuppression or lowering biological barriers, these microorganisms cause serious infections. Among *Candida* species, *C. albicans* is the species most associated with superficial and systemic infections (Pfaller and Diekema, 2010). Among the other species, *C. auris*, has emerged as a major threat due to its remarkable tendency for intrinsic multidrug-resistance (Kordalewska and Perlin, 2019). Treatment of invasive candidiasis is based on three classes of antifungals: polyenes, azoles and echinocandins. However, these drugs have variable effectiveness in the setting of biofilms (Tumbarello et al., 2007; Sawant and Khan, 2017).

Diverse NPs have been studied for their activity against *Candida*. For example, gold NPs (AuNPs) have been studied aiming at their antifungal activity in *C. albicans* biofilms, since in conjunction with photosensitizer, AuNPs can increase the effectiveness of photodynamic therapy (Khan et al., 2012; Sherwani et al., 2015; Maliszewska et al., 2017). AuNPs can destabilize the cell membrane of the pathogen through direct interaction with proteins and lipids. In addition, the association of photosensitizers with metallic nanoparticles can reduce the risk of pathogens developing resistance to photodynamic therapy (Maliszewska et al., 2017).

The effect of AgNPs against *Candida* spp. have also been widely studied, both against planktonic cells and biofilms (Monteiro et al., 2011; Lara et al., 2015). Kischkel et al. (2020) evaluated the efficacy of AgNPs carried with propolis extract (PE) against mature biofilms of *Candida* species and other fungi and observed that the concentration required for the fungicidal activity of the formulation was below the cytotoxic concentration.

Curcumin has broad antimicrobial activity and it is nontoxic. However, due to several factors, such as degradation and rapid systemic elimination, curcumin has had limited applications as a therapeutic due to its low bioavailability in the blood (Anand et al., 2007). AgNPs have been created to enhance curcumin delivery. The Curcumin—AgNPs significantly inhibit fluconazole resistant *C. albicans* and *C. glabrata*, and the inhibition depended on the concentration of curcumin used (Paul et al., 2018).

Rózalska et al. (2018) studied biogenic AgNPs against reference strains of *C. albicans, C. glabrata*, and *C. parapsilosis* and found that the particles were effective at a low minimum inhibitory concentration (MIC) range (1.56–6.25 μg/mL), which did not concomitantly show cytotoxicity. Notably, the biogenic AgNPs were stable *in vitro* for long periods. In addition, AgNPs used in combination with fluconazole were able to decrease the biological activity of *C. albicans* biofilms, which was attributed to the ability of the NPs to enhance the penetration of the azole disturbing the cell membrane, while Ag destabilized efflux transporter efficacy. However, despite the advantages of biomolecule synthesis, AgNPs have a tendency to aggregate and this effect impairs their antimicrobial activity (Rózalska et al., 2018).

Vazquez-Muñoz et al. (2014) observed that AgNPs did not penetrate the intracellular environment and that fungal cell death may have occurred due to the release of silver ions from AgNPs accumulated outside the cells, resulting in smaller NPs located throughout the cytoplasm. In another publication, ROS production, changes in ergosterol levels, and other effects acted together to enhance the effect of AgNPs against *C. albicans* (Radhakrishnan et al., 2018b). In addition, intracellular ROS levels could be reversed by ascorbic acid antioxidant without altering the effectiveness of AgNPs against *C. albicans* (Radhakrishnan et al., 2018a).

The antifungal effects of AmB and nystatin coupled to magnetic NPs (MNP-AmB and MNP- Nystatin) have been studied against clinical isolates of *C. albicans*. Niemirowicz et al. (2016) observed that MNP-AmB and MNP-Nystatin showed significant fungicidal effect and prevented biofilm formation. The observed effect could be due to catalase inactivation (Cat1) in cells exposed to nanosystem treatment, since disturbance of the redox balance could lead to inhibition of *C. albicans* growth. Subsequently, magnetic NPs coated with peptide LL-37 and ceragenin CSA-13 also showed fungicidal effects against *Candida* sp. due to increased ROS production associated to pore formation in the cell membrane, thus assisting NPs penetration into the yeast cells (Niemirowicz et al., 2017b).

Using encapsulated AmB in PLGA-PEG NPs (PLGA-PEG-AmB), the efficacy, toxicity, and oral bioavailability of these formulations were evaluated, *in vivo* and *in vitro*. Compared to free AmB, the PLGA-PEG-AmB NPs decreased MIC against *C. albicans* cells. Using a hemolysis assay, NP formulations had lower toxicities compared with Fungizone®. *In vivo*, blood urea nitrogen, and plasma creatinine measurements remained normal after a week of oral administration of PLGA-PEG-AmB NPs in rats. Finally, bioavailability of the PLGA-PEG-AmB NPs was further enhanced with the addition of glycyrrhizin acid (GA) (Radwan et al., 2017).

NPs of PLGA with chitosan containing AmB (PLGA-CHI-AmB) were synthesized and the derived NPs achieved nanometer size, low polydispersity, positive surface charge, and good encapsulation capacity for AmB. Notably, chitosan, in addition to having mucoadhesive properties, helps maintain the stability of nanoparticles and increases their biocompatibility. However, the PLGA-CHI-AmB showed variable MICs against with different *Candida* sp.. This result can be explained by the prolonged release of AmB, resulting in less activity *in vitro*. Although AmB is available to act on the target, the release kinetics of the NPs of PLGA with chitosan needs to be more controlled to achieve efficacy (Ludwig et al., 2018).

Among the virulence factors of *C. albicans*, the transition from yeast to hyphae represents an important factor in its pathogenicity. Farnesol is a molecule produced by *C. albicans* and it is an important quorum-sensing molecule that inhibits the growth of hyphae (Kruppa, 2009). Chitosan NPs were formulated to encapsulate farnesol and miconazole, which were then evaluated in a murine model of vulvovaginal candidiasis. Interestingly, no *in vitro* synergism between miconazole and farnesol was found. Farnesol-containing chitosan NPs, however, were effective in reducing the pathogenicity in mice, and farnesol-containing NPs inhibited hyphal growth in *C. albicans*. Additionally, the NPs tested showed no toxicity in cultured fibroblasts (Fernandes Costa et al., 2019).

SLNs and NLCs were created that were easily loaded with AmB and the NPs displayed lower hemolytic activity compared with Fungizone®. The AmB SLNs and NLCs were also more effective than free AmB or Fungizone® against *C. albicans*. The data suggest that these formulations may increase antifungal activity, increase AmB solubility, and decrease the toxic effect of treatment. This effect may be due to by the sustained release of AmB in the formulations and by its monomeric state, since AmB with a low degree of aggregation is more selective and binds mainly to ergosterol (Jansook et al., 2018).

Antifungal SLNs have been studied with drug resistant *Candida*. Fluconazole loaded SLNs (FLZ-SLNs) were more effective than free fluconazole against the species tested. The FLZ-SLNs displayed fast drug release in the first 30 min followed by sustained release over 24 h (Moazeni et al., 2016; Kelidari et al., 2017). One of the main resistance mechanisms in yeasts is the overexpression of efflux pumps, reducing the levels of azoles within the cell. The increased susceptibility to antifungals, in this case, may be related to the protection that NLCs provided to FLZ, protecting the drug from being discharged from the cell. In addition, the hydrophobic surface of FLZ-NLCs can increase the penetration of the drug into yeast (Kelidari et al., 2017).

Lipid core NPs and fluconazole containing NLCs were evaluated in fluconazole resistant *Candida*. Although the NLCs were not effective, the lipid nucleus NPs were active at reduced fluconazole concentrations. Additionally, the lipid nucleus-fluconazole NPs prevented fluconazole recognition by efflux pumps in fungal cells (Domingues Bianchin et al., 2019).

To reduce the toxicity reported for miltefosine and maintain its antifungal effect, Spadari et al. (2019) evaluated the activity of miltefosine-loaded alginate NPs against *Candida* and *Cryptococcus* species. Miltefosine encapsulation in 80% alginate NPs significantly reduced the toxic effects compared to free miltefosine in an *in vitro* system as well as in *Galleria mellonella*. Moreover, the treatment of *G. mellonella* infected with *C. albicans* with miltefosine -alginate NPs significantly extended larval survival time. The effect obtained may be associated with the controlled release of the drug, since alginate-based nanocarriers allow for the constant release of the drug, which can maintain its bioavailability and reduce potential adverse effects. Another advantage described is the size of the nanoparticles obtained in this study (average size of 279.1 ± 56.7 nm), which are favorable for mucosal and oral administration (Spadari et al., 2019).

The first study involving NPs as a *C. albicans* vaccine was published by Han and Cutler (1995), in which they used phosphatidylcholine and cholesterol liposomes to carry manganese extracted from *C. albicans*. Vaccination provided protection against widespread infection and the antiserum from infected animals was able to protect BALB/cByJ and SCID mice against *C. albicans* and *C. tropicalis*. Also, in this study, a specific monoclonal antibody was obtained, MAb B6.1, which protected against widespread infection. Subsequently, the vaccine potential of this mAb against vaginal candidiasis was evaluated (Han et al., 1998). Concurrently, another study evaluated vaccine potential of *C. albicans* ribosomes incorporated into liposomes composed of dimyristoyl phosphatidyl choline (DMPC) and dimyristoyl phosphatidyl glycerol (DMPG). Immunization of mice with these liposomes resulted in 60% survival rate of animals with disseminated candidiasis (Eckstein et al., 1997).

Heat shock protein 90 represents a highly conserved *C. albicans* chaperone that is also an immunogenic protein abundantly present in the fungal cell wall, which has been studied as a potential vaccine candidate (Matthews et al., 1987). Mašek et al. (2011), incorporated rHSP90 into the surface of nickel chelating liposomes associated with norAbuMDP pyrogen adjuvant, a compound of lipophilic derivatives of muramyl dipeptide (MDP), for intradermal vaccination of BALB/c mice and observed comparable Th1 and Th2 response to Freund's complete adjuvant vaccine. Later, Knotigová et al. (2015), evaluated the vaccine efficacy of rHSP90 in nickel-chelating liposomes associated with two pyrogen-free adjuvants (norAbuMDP and norAbuGMDPs) in ICR mice and rabbits, and showed stimulation of innate and adaptive immune response against the rHSP90-containing nano formulation.

More recently, Carneiro et al. (2015, 2016) employed dimethyldioctadecylammonium bromide (DODAB) monoolein-based liposomes for delivery of *C. albicans* wall proteins. In the first study, prophylactic vaccination using the NPs in BALB/c mice stimulated humoral and cellular immune response with production of IgG antibodies against two specific proteins found in the cell wall, Cht3p and Xog1p. Additionally, there was no apparent toxicity of the NPs. In a second study, two formulations with different lipid concentrations for protein loading, called ADS1 and ADS2, each containing a total lipid concentration of 1,774 and 266 μg/ml, respectively, were evaluated. The results showed that only the administration of ADS1 was able to confer protection against infection in mice, with a high production of specific antibodies that increased fungal phagocytosis. There was also an increased production of IL-4, IL-17, and IL-10 cytokines, demonstrating a mixed Th1, Th2, and anti-inflammatory response.

Studies involving NPs for treatment of candidiasis are shown in Table 1 and for vaccination in **Table 4**.

**Table 1:** Nanoformulations studied for the treatment of fungal infections caused by *Candida* and *Cryptococcus* yeasts.

**Nanoparticle**	Drug ^(^*^)^	**Fungi**	***In vitro*/*in vivo***	**References**
AuNP	–	*C. albicans*	*in vitro* and *in vivo* (mice)	Khan et al., 2012; Sherwani et al., 2015; Maliszewska et al., 2017
AgNP	FLZ	*C. albicans* *C. glabrata* *C. parapsilosis* *C. tropicalis* *Candida kefyr*	*In vitro*	Monteiro et al., 2011; Vazquez-Muñoz et al., 2014; Lara et al., 2015; Paul et al., 2018; Radhakrishnan et al., 2018a,b; Rózalska et al., 2018
	Propolis	*C. albicansC. glabrataC. parapsilosisC. tropicalisC. kruseiF. oxysporumT. interdigitaleT. rubrumM. canis*	*In vitro*	Kischkel et al., 2020.
Magnetic	AmB/NYS	*C. albicans*	*In vitro*	Niemirowicz et al., 2016, 2017b.
PLGA-PEG PLGA-CHI	AmB	*C. albicans* *C.glabrata* *C. tropicalis* *Trichosporon asahii* *C. guilhermondii*	*In vitro* and *in vivo* (rats)	Radwan et al., 2017; Ludwig et al., 2018
Chitosan	farnesol/miconazole	*C. albicans*	*In vitro* and *in vivo* (mice)	Fernandes Costa et al., 2019
Solid lipid	AmB/FLZ	*C. albicans* *C. glabrata* *C. parapsilosis* *C. neoformans* *fumigatus* *Penicillium marneffei*	*In vitro*	Moazeni et al., 2016; Jansook et al., 2018
Nanostructured lipid carrier	AmB/FLZ	*C. neoformans* *C. tropicalis* *C. krusei* *C. paraposilosis* *C. glabrata* *C. kefyre* *fumigatus* *Penicillium marneffei*	*In vitro*	Kelidari et al., 2017; Jansook et al., 2018; Domingues Bianchin et al., 2019
Core-shell architecture of silver nanostructure (Pd@AgNSs)	AmB	*Cryptococcus spp*.	*In vitro*	Zhang C. et al., 2016
AgNPs and AuNPs	–	*C. neoformans* *C. gattii* *Candida* spp. *Dermatophytes*	*In vitro*	Ishida et al., 2014; Rónavári et al., 2018
Chloroaluminum phthalocyanine nanoemulsion (ClAlP/NE)	–	*C. neoformans*	*In vitro* photodynamic antimicrobial chemotherapy (PACT)	Rodrigues et al., 2012
PLA-b-PEG coated with polysorbate 80 (Tween-80)	AmB	*C. neoformans*	*In vivo*	Ren et al., 2009
Polybutylcyanoacrylate (PBCA)	AmB	*C. neoformans*	*In vivo* (mice)	Xu et al., 2011
Angiopep-PEG-PE polymeric micelles	AmB	*C. neoformans*	*In vitro* and *in vivo* (mice)	Shao et al., 2012
BSA nanoparticles coated with polysorbate- 80	AmB	*C. neoformans*	*In vitro*	Pedroso et al., 2018
Nanoparticle crystal encapsulated (encochleated)	AmB/5FC	*C. neoformans*	*In vivo* (mice)	Lu et al., 2019
PLGA/PLGA-PEG	ITZ/AmB	*C. neoformans* *C. albicans*	*In vitro* and *in vivo* (mice)	Moraes Moreira Carraro et al., 2017; Tang et al., 2018
SDCS nanomicelles	AmB	*C. neoformans, C. albicans*	*In vitro*	Usman et al., 2018
PAMAM-sulfonamide dendrimers	-	*C. neoformans* *C. glabrata*	*In vitro*	Carta et al., 2015

(^*^) * Drugs: AmB, Amphotericin B; ITZ, Itraconazole; NYS, Nystatin; FLZ, Fluconazole; 5FC, 5 fluorocytosine*.

### Cryptococcosis

Cryptococcosis is a systemic mycosis caused by *C. neoformans*/*C. gattii* species complexes (Hagen et al., 2015), associated with high morbidity and mortality rates, especially in immunocompromised individuals and low-income countries (reviewed in Mourad and Perfect, 2018). The infection begins with inhalation of fungal propagules and in healthy individuals can be eliminated without significant symptoms. Asymptomatic spread frequently occurs, which can also lead to disease relapse in immunosuppressed infected individuals. Immunocompromised hosts can develop the primary infection. In either situation, the mycosis frequently affects the central nervous system as a meningoencephalitis (Kwon-Chung et al., 2014). Although individuals infected with human immunodeficiency virus (HIV) are the main risk group affected by *C. neoformans*, patients receiving immunosuppressive drugs and chemotherapy are also at risk (Sloan and Parris, 2014). Notably, *C. gattii* is mostly associated with immunocompetent individuals, although some other risk factors may contribute to the development of the disease (Marr et al., 2012; Chen et al., 2014; Saijo et al., 2014).

The choice treatment of cryptococcosis presented as cryptococcal meningitis or severe pulmonary cryptococcosis, is based on the administration of AmB in combination with 5-fluorocytosine, followed by fluconazole as a maintenance drug, for weeks to lifetime (Perfect et al., 2010). In countries where 5-fluorocytosine is not available fluconazole can be used as a replacement in conjunction with AmB (Perfect et al., 2010). In little resourced areas, high doses fluconazole may be used as primary therapy.

AmB deoxycholate remains an important drug for the treatment of deep fungal infections. However, its use for the treatment of cryptococcal meningitis is limited due to the inability of the drug to cross the blood-brain barrier (Xu et al., 2011). Searching for a brain drug delivery system, some nanocarriers have been studied and interesting results against *Cryptococcus* sp. have been reported (Ren et al., 2009; Xu et al., 2011; Pedroso et al., 2018). Early nannocarrier studies used polysorbate 80, a surfactant and emulsifier that improves NP uptake by human and bovine primary brain capillary endothelial cells. Polysorbate 80 coated particles can increase the concentration of drug in the brain by up to 20 times 1 hour after the injection and are therefore considered an efficient brain “driver” (Ramge et al., 2000). Xu et al. (2011) developed AmB-polybutylcyanoacrylate NPs and polysorbate coated (AmB-PBCA-NPs) for systemic administration in a mouse model. According to the authors, NPs of ~69 nm were detected in the brain 30 min after injection and in a higher concentration than liposomal AmB. Interestingly, AmB deoxycholate was not detected in the brain; however, survival rates were 80, 60, and 0% for AmB-PBCA-NPs, Liposomal AmB, and AmB deoxycholate, respectively. According to Ren et al. (2009), polysorbate 80 also improves the trapping effectiveness of AmB in the polymeric system as PLA-b-PEG. In *in vitro* tests, 100% of the AmB was released between 35 and 40 h. In NPs containing the polysorbate, almost 100% of AmB was released between 60 and 70 h.

Lipid based AmB cochleates (CAMB) is a new type of AmB nanocarrier with potential for oral administration, showing greater stability and resistance to gastrointestinal degradation (Santangelo et al., 2000). Lu et al. (2019) recently studied the administration of CAMB in combination with 5-fluorocytosine and found it to be highly effective in a murine model of cryptococcal meningoencephalitis. *In vivo* data also showed that CAMB doses up to 90 mg/kg/day appeared to be non-toxic. A particular benefit of the CAMB formulation is that the release of the drug is calcium dependent and can thus maintain its stability until reaching the intracellular environment. This provides a mechanism for controlling drug release as well as enhancing CNS drug levels (Lu et al., 2019).

Several studies, therefore, have focused on the development of safe, effective, and less expensive alternatives for the use of AmB. Liposomal, colloidal dispersion, and lipid complexes are examples of nano formulations that have been shown to attenuate toxic effects in therapy (Reviewed in Spadari et al., 2017).

Formulations with nanomicelles of AmB using sodium deoxycholate sulfate (SDCS) have been developed for targeted pulmonary delivery through inhalation of nanoformulation. According to Usman et al. (2018), the AmB-SDCS is equivalent in efficacy to Fungizone®, but the NPs does not cause toxic effects in respiratory and kidney cell lines. AmB-SDCS formulations showed activity against *C. neoformans, C. albicans*, and *S. cerevisiae*. A phagocytosis assay using NR8383 cells revealed that AmB-SDCS accumulated within the host effector cells without evidence of phagocytic cell damage. As we have already discussed, macrophages internalize particles of 0.5–5 μm. AmB-SDCS have a diameter of 0.9–1.6 μm. In addition to size, the surface chemistry of NPs can also influence uptake by macrophages as hydrophobic particles can stick to the cell surface (Xiang et al., 2006; Usman et al., 2018). This study demonstrated the effectiveness of an aerosolized lipid formulation in the delivery of AmB to alveolar macrophages *in vitro*, one of the main reservoirs of fungi such as *Cryptococcus* and *Aspergillus*. However, further studies are needed to validate the method *in vivo*.

Nanotechnology can overcome certain limitations of current antifungal drugs (Niemirowicz et al., 2017a). Similar to AmB, the hydrophobic character of ITZ causes the drug to have poor tissue penetration. Nanocarriers used for controlled drug release could help increase ITZ levels. Curić et al. (2017) incorporated ITZ into poly (butyl cyanoacrylate) nanocapsules, helping the drug stability and targeting. Aiming at oral administration, a system using PLGA and chitosan NPs was developed and analyzed for efficacy against *C. neoformans* pulmonary infection. A chitosan-binding peptide, screened by phage display, was conjugated to PLGA NPs (CP-NPs) with or without free chitosan (C-CP-NPs) and ITZ was incorporated in the NP. Notably, free chitosan (C-CP-NPs/ITZ) did not influence the efficiency of drug incorporation and it did not impact drug release. Both CP-NPs/ITZ and C-CP-NPs/ITZ prolonged the survival of mice with pulmonary cryptococcosis, although C-CP-NPs/ITZ was more effective (Tang et al., 2018).

Antimicrobial activity of some nanomaterials, such as Ag and Au, is commonly reported, inhibiting or killing both eukaryotic and prokaryotic pathogens (Musarrat et al., 2010; Yu et al., 2016). Although safety uncertainty of AgNPs causes conflicting information, the therapeutic effect against some pathogens is unquestionable (Vazquez-Muñoz et al., 2017).

Zhang C. et al. (2016) conducted a study using core-shell architecture of Ag nanostructure (Pd@AgNSs), to evaluate the antifungal activity against invasive fungi. This nanostructured Ag was obtained through deposition techniques based on palladium seeds that generated NPs with a high degree of biocompatibility due to the uniform size and shape. Pd@AgNSs displayed broad activity against ascomycetes and basidiomycetes, including strains considered resistant to fluconazole. The antifungal effect of the Pd@AgNSs was independent on the size of the nanoparticles. The authors speculate that the hexagonal shape of the NPs had a greater influence on antifungal properties and that this potent activity masked the expected size effects. Pd@AgNSs were cidal to fungi through mechanisms that included alterations in protein synthesis and energy metabolism. In addition, Pd@AgNSs induced increased numbers of vacuoles, which may be associated with survival strategies of the fungus itself to improve protein transport, since cell stress can increase energy demand and therefore result in increased numbers of mitochondria. Pd@AgNSs also acted synergistically with AmB, reducing the effective AmB concentration to 0.125 μg/mL.

NPs synthesized by biological routes have been explored for efficacy against cryptococcosis. In this type of synthesis, we emphasize that it is necessary to take into account the source of obtaining secondary metabolites. The source must offer a toxin-free extract that is rich in substances such as flavonoids, terpenoids, among others, which are mainly responsible for the synthesis and stabilization of metal ions that influence the final cytoxicity of the nanoformulation (Ahmed et al., 2018; Lakshmeesha et al., 2019).

Ag and Au NPs were synthesized using cell-free extract of *Phaffia rhodozyma*, the red yeast containing astaxanthin, a type of natural antioxidant that aids in the formation of metallic NPs. These biologically synthesized AgNPs and AuNPs showed no toxicity to human HaCat keratinocytes. Although the AgNPs were broadly effective against basidiomyces and ascomyces, the AuNPs were also able to inhibit *C. neoformans* (Rónavári et al., 2018). Ishida et al. (2014) created Ag nanostructures using an aqueous extract of *Fusarium oxysporum* that was effective against *Candida* and *Cryptococcus* species, particularly *C. gatti*. The AgNPs induced changes in the cytoplasmic membrane and wall of *Cryptococcus* spp. strains, but not of *Candida* spp..

Photodynamic antimicrobial chemotherapy is a method that can be combined with nanocarriers (Rodrigues et al., 2012). In this case, the nanocarrier is called a photosensitizer and can be applied to the skin lesion caused by the fungus where it can bind fungal cells and accumulate at the infection site. The photosensitizer is then exposed to visible light at appropriate wavelengths to induce the production of ROS resulting in the death of the fungus (Donnelly et al., 2008). Therefore, the photosensitizer must be an agent that is directed at the fungal cell while the light focuses on the lesion. Photodynamic antimicrobial therapy was evaluated in melanized *C. neoformans* cells using chloroaluminum phthalocyanine incorporated into NE (ClAlP/NE). ClAlP/NE effected the viability of *C. neoformans* cells in a dose depenent manner according to both the amount of the particle and the intensity of the light applied. The use of this alternative was helpful in the treatment of skin lesions caused by *C. neoformans* and other fungi (Rodrigues et al., 2012).

The studies involving NPs for cryptococcosis treatment are summarized in Table 1.

### Aspergillosis

The members of the genus *Aspergillus* are described as opportunistic pathogens capable of inducing allergic reactions to systemic infections in humans. *A. fumigatus* is the most predominant species, responsible for 90% of invasive infections (Paulussen et al., 2017). It is a globally ubiquitous organism and dominant in different habitats due to various morphological and physiological factors (Cray et al., 2013). Importantly, azole resistance is an emerging problem in *A. fumigatus*, which has resulted in treatment failures (Seyedmousavi et al., 2014).

Different types of polymeric NPs have been explored as carriers of AmB for the treatment of experimental aspergillosis (Shirkhani et al., 2015; Salama et al., 2016; Yang et al., 2018). Among them, Italia et al. (2011) reported the efficacy of PLGA NPs for oral administration of AmB, The oral administration of PLGA NPs was superior to parenteral Ambisome® and Fungizone® in neutropenic murine models of disseminated and invasive aspergillosis. Notably, conventional AmB (Fungizone®) is ineffective in this model. The AmB PLGA NPs promoted greater oral absorption of AmB compared to AmB alone. The NPs were able to protect the drug from degradation by pH and gastrointestinal enzymes, thereby overcoming incoming metabolism and allowing more NPs to be captured by lymph nodes (Italia et al., 2011). Therefore, oral administration of AmB may represent a promising strategy for the treatment of disseminated fungal infections, or at least azole refractory oral thrush. Similarly, Van de Ven et al. (2012) found that a PLGA and nanosuspension NPs containing AmB administered by intraperitoneal route in mice were two and four times more effective in reducing fungal load, respectively, than Ambisome® and Fungizone® in disseminated aspergillosis models. In this case, the authors hypothesized that the state of aggregation of AmB in the delivery system may influence the interaction of NPs with ergosterol present in fungal membranes. These differences in the aggregation states of the particles in solution were confirmed by analyzing the UV/VIS spectra of the evaluated formulations. In addition, the authors speculated that PLGA and nanosuspension NPs may have transported the drug directly to the tissue compartment, since the nanoformulation has an ideal size for blood circulation (≤100 nm) and may promote rapid uptake by the reticuloendothelial system, as is the case of formulations like Abelcet® and Amphocil® (Van de Ven et al., 2012).

Some nanoformulations, besides being effective in the treatment of aspergillosis *in vitro* and *in vivo*, may have reduced side effects in relation to some commercially available formulations. mPEG-b-P(Glu-co-Phe) carrying AmB is stable in plasma and has lower nephrotoxicity than free AmB (Yang et al., 2018). A PEG-Lipid NPS carrying AmB showed low cytotoxicity against human kidney cells than Fungizone® and AmBisome®, in addition to lower hematotoxicity compared to Fungizone® (Jung et al., 2009). PEG/PLA with ITZ caused moderate hemolysis, although it showed superior *in vitro* antifungal activity compared to free ITZ (Essa et al., 2013). The increased toxicity of Fungizone® or free ITZ can be explained by the faster release of the drug compared to studied NP and/or Ambisome® formulations. The strong interactions between AmB and the lipids, phospholipids or polymers present in the these formulations can delay the release of the drug, and, consequently, reduce the cytotoxic effects. On the other hand, a lower toxicity of PEG-LNPs compared to AmBisome® suggests that nanoformulation may be more efficient (Jung et al., 2009).

Some studies have considered evaluating the efficacy of inhaled formulations in the prophylaxis of aspergillosis, since infection by Aspergillus sp. starts from inhalation of infectious spores (Rodríguez-Cerdeira et al., 2014). A lipid complex of AmB (Abelcet®) was administered as an aerosol for prophylaxis for pulmonary aspergillosis model in rats. Through this technique it was possible to observe higher and prolonged levels of the compound in the lungs, and higher survival rates after 2 and 10 days of infection compared to aero-AmB (Fungizone®) (Cicogna et al., 1997). In 12 human lung transplant recipients, the nebulized Abelcet® was well distributed in the lungs, but the deposition rate was below expectations (Corcoran et al., 2006). Shirkhani et al. (2015) explored the efficacy of PMA, delivered via nebulizer to prevent *Aspergillus* infection in a mouse transplant immunosuppression model, in which 3 days of prophylactic treatment were sufficient to deposit the AmB NPs in the lung and prevent fungal growth. In this case, a polymethacrylic acid was used to transform the insoluble AmB into a 78–9 nm particle of water-soluble AmB-PMA and with a UV/VIS spectrum identical to the liposomal AmB. PMA does not have immunomodulatory properties, so the administration of AmB-PMA by nebulization would constitute a pre-transplant prophylactic therapy approach capable of effectively delivering the drug to the lung and protecting against the development of fungal infections that initially come into contact with the lung.

Different AmB formulations have been tested to treat eye complications caused by *A. fumigatus* (Zhao et al., 2015; Khames et al., 2019). The development of nanostructured systems for delivering medication to the cornea consists of advantages such as improving the penetration of the drug into the cornea, improving mucoadhesive properties and prolonged residence time. SLNs represent an efficient delivery system for this purpose due to the lipophilic nature and the small size that allows the penetration of physiological barriers and the sustained release of drugs without impairing vision. In order to improve the penetration of natamycin (NAT) into the cornea, Khames et al. (2019) incorporated the drug into SLNs. The NAT-SLNs effectively released NAT for 10-h and improved the corneal permeation compared to a free drug. The NAT-SLNs were more potent than free NAT *in vitro*. Furthermore, the NAT-SLNs showed no cytotoxic effect in corneal tissues obtained from goats.

On the other hand, Zhao et al. (2015) compared voriconazole and liposomal AmB in guinea pig endophthalmitis model. Both drugs were able to treat endophthalmitis. However, voriconazole was more effective than liposomal AmB using a similar dose (20 μg) in the initial treatment period, since the group treated with voriconazole after induction of endophthalmitis, showed lower inflammation in the early and middle stages. The retinal histopathology was normal after administration of both drugs. A lower performance of liposomal AmB in the initial stage of treatment can be explained due to the presence of cholesterol, acting as a stabilizer in NPs, as well as the controlled release of the drug that occurs when the fungus comes into contact with liposomal AmB and the drug is released liposome. Thus, resulting in a delayed efficacy compared to free voriconazole.

Nanoformulations for AmB delivery are the most studied, considering that it is a first line antifungal in the treatment of fungal infections. Side effects of AmB deoxycholate have been reduced with the development of several liposomal formulations. However, these formulations are not produced under the same conditions and/or in the same concentration of lipids and drugs, for example, as mentioned in topic 2.2 on the composition of Abelcet® and Ambisome®. On the other hand, two formulations with similar chemical composition can result in particles of different sizes, such as AmBisome® (77.8 nm) and Lambin® (122.2 nm). These differences can influence the physical-chemical properties as well as the biological activity of these formulations (Olson et al., 2015). AmBisome®, for example, is one of the most commonly reported AmB formulations referenced in articles to compare the efficiency of other nanoformulations (Clemons et al., 2005; Jung et al., 2009; Sheikh et al., 2010; Italia et al., 2011). Below, we cite some articles that show some differences in the biological activity of these commercial liposomal formulations that can be explained by differences in the synthesis and composition of the final formulation.

A study compared the toxicity and efficacy of two AmB lipid formulations, AmBisome® and Lambin®, in mice. The application of a single dose of 50 mg/kg of the drugs led to 80% mortality with Lambin® and 0% with AmBisome®. After daily intravenous administration of 5 mg/kg of the drugs, tubular renal changes were observed in mice that received Lambin®. Although both drugs significantly decreased fungal burden in the lungs of mice treated after *A. fumigatus* infection, survival rates were 30% with Lambin® and 60% with AmBisome®. The histopathology showed that treated animals with AmBisome® presented fewer fungal elements and less tissue damage (Olson et al., 2015).

Olson et al. (2006) established the ideal concentration for treatment of pulmonary aspergillosis taking into consideration the toxicity and efficacy of AmBisome® and Abelcet® formulations in a murine model. Both formulations showed prolonged survival at 12 mg/kg. Due to the reduced nephrotoxicity of AmBisome®, increased doses of 15 or 20 mg/kg can be used safely. Seyedmousavi et al. (2013) demonstrated that AmBisome® is able to prolong the survival of the mouse regardless of the mechanism of azole resistance displayed in isolates used for infection.

Lewis et al. (2007) compared the accumulation kinetics of AmBisome® and Abelcet in the lungs of immunosuppressed mice and with invasive pulmonary aspergillosis. In conclusion, Abelcet® at 5 mg/kg/day conveys active AmB in the lung faster than AmBisome®, leading to a more rapid reduction in fungal burden. At concentrations higher than 10 mg/kg/day there was no pharmacodynamic difference between the formulations. Regarding neutropenia, Siopi et al. (2019), demonstrated in mice that the appropriate doses of AmBisome® range 1–3 mg/kg for non-neutropenic patients and 7.5–10 mg/kg for neutropenic patients with isolates of *A. fumigatus* with MIC from 0.5 to 1 mg/L.

Patients were evaluated for responsiveness to AmBisome® in chronic pulmonary aspergillosis therapy. Seventy-one patients were included in the study, in which all responded to long-term therapy; however, 25% patients developed acute kidney injury, indicating that these drugs should be used with caution (Newton et al., 2016).

Combination therapy for the treatment of invasive fungal infections can be explored in an attempt to lessen the side effects of more potent drugs like AmB by combining it with other less toxic antifungals. Thus, promoting the reduction of the concentration of AmB used in a monotherapy. In addition, some studies based on the combination of antifungals aim to assess whether there is synergistic or additive potential between specific antifungals (Olson et al., 2010). In murine models of disseminated aspergillosis, combined therapy of AmBisome® prior to echinocandin or both drugs administered together were as effective as AmBisome® alone. Both classes of drugs target the cell membrane, as we mentioned earlier. The authors support the use of AmBisome® before echinocandins due to the greater reduction in fungal burden observed in the study (Olson et al., 2010). The combination of AmBisome® with voriconazole was effective for treatment of CNS aspergillosis, while the combination of AmBisome® with micafungin or caspofungin did not show much benefit in CNS disease treatment (Clemons et al., 2005).

The oligosaccharide OligoG, an alginate derived from seaweed, inhibited the growth of *Candida* and *Aspergillus* strains *in vitro*, in a dose dependent manner. In addition, it inhibited hyphal growth depending on the strain and disrupted biofilm formation. OligoG was also associated with other antifungals such as nystatin, AmB, fluconazole, miconazole, voriconazole and terbinafine, which potentiated their inhibitory effects *in vitro*. The combination of drugs led to a decrease of up to 4 times in MIC, with nystatin being the best association, promoting a reduction of up to 16 times in MIC (Tøndervik et al., 2014).

Salama et al. (2016) evaluated the activity of cross-linked chitosan biguanidine (CChG) loaded with AgNPs. The thermal stability of the polymer was improved due to silver incorporation, resulting in NPs with lower cytotoxicity for MCF-7 cells (human breast adenocarcinoma cell line) and improved antimicrobial activity against bacteria and fungi compared to chitosan or CChG. In this case, the association of polymers with AgNPs is mainly aimed at improving antimicrobial activity due to the intrinsic properties of Ag in association with sustained delivery through polymers. The degradation of the nanocomposite at higher temperatures after the association of CChG and AgNPs, is due to the interaction of these compounds that promoted an enhanced stabilization of the structure.

The studies involving NPs in aspergillosis treatment are summarized in Table 2.

**Table 2:** Nanoformulations studied for the treatment of fungal infections caused by the *Aspergillus* sp..

**Nanoparticle**	Drug ^(^*^)^	**Fungi**	***In vitro/in vivo***	**References**
PLGAvv	AmB	*A. fumigatus*	*In vivo* (mice)	Italia et al., 2011; Van de Ven et al., 2012
	VOR		*In vivo* (rabbits)	Yang et al., 2011
Chitosan biguanidine	Silver	*A. fumigatus* *G. candidum* *S. recemosum*	*In vitro*	Salama et al., 2016
PMA	AmB	*A. fumigatus*	*In vitro/in vivo* (mice)	Shirkhani et al., 2015
PEG/PLA	ITZ	*A. fumigatus* *C. albicans*	*In vitro*	Essa et al., 2013
PEG-LNPs	AmB		*In vitro/in vivo* (rats and mice)	Jung et al., 2009
Alginate oligosaccharides (OligoG)	–	*C. albicans* *C. parapsilosis C. Krusei* *C. lusitaniae* *C. tropicalis* *C. glabrata* *niger* *A. fumigatus* *A. flavus*	*In vitro*	Tøndervik et al., 2014
mPEG-*b*-P-(Glu-*co*-Phe))	AmB	*A. fumigatus*	*in vivo* (mice)	Yang et al., 2018
Nanossuspension	AmB	*C. albicans* *A. fumigatus* *T. rubrum*	*In vitro/in vivo* (mice)	Van de Ven et al., 2012
	ITZ	*A. fumigatus*	*In vivo* (quails)	Wlaz et al., 2015
Liposomal (AmBisome®)	AmB	*A. fumigatus*	*In vivo* (mice)	Clemons et al., 2005; Olson et al., 2006, 2010, 2015; Lewis et al., 2007; Jung et al., 2009; Italia et al., 2011; Seyedmousavi et al., 2013; Siopi et al., 2019
			*In vivo* (rabbits and mice)	Sheikh et al., 2010.
			*In vivo* (human)	Newton et al., 2016; Godet et al., 2017
Lipossomal	AmB	*A. fumigatus*	*In vitro/in vivo* (guinea pig)	Zhao et al., 2015
Liposomal—Lambin® (Lbn)	AmB	*A. fumigatus*	*In vivo* (mice)	Olson et al., 2015
Lipossomal (Abelcet®)	AmB	*A. fumigatus*	*In vivo* (mice)	Olson et al., 2006; Lewis et al., 2007
			*In vivo* (rats)	Cicogna et al., 1997
SLNs	NAT	*A. fumigatus* and *C. albicans*	*In vitro/ex vivo* (goat corneas)	Khames et al., 2019

(*) * Drugs: AmB, Amphotericin B; VOR, voriconazole; ITZ, Itraconazole; NYS, Nystatin*.

### PCM

PCM is a systemic mycosis caused by thermo-dimorphic fungi of the genus *Paracoccidioides* (Taborda et al., 2015). PCM has two clinical forms, acute/subacute form (juvenile) and chronic form (adult) (Shikanai-Yasuda and Mendes, 2007). PCM treatment is based on chemotherapy, the chief ones being azole agents such as fluconazole, polyenes such as AmB and sulfonamides such as Bactrim® (Shikanai-Yasuda and Mendes, 2007; Amaral et al., 2009; Souza and Amaral, 2017).

The targeting of NPs commonly can be achieved by adding antibodies to the surface of the particle. However, additional molecules also have this potential to target NPs to specific tissues. Just as polysorbate 80 assists in targeting drugs to the brain, the incorporation of dimercaptosuccinic acid (DMSA) in nanocarrier systems directs NPs mainly to the lung. However, the mechanisms by which this tropism occurs has not been well-established (Amaral et al., 2009). PLGA and DMSA NPs carrying AmB (Nano-D-AMB) were evaluated for treatment efficacy of chronic PCM caused by *P. brasiliensis*. After 30 days of infection, BALB/c mice were treated with 6 mg/kg Nano-D-AMB at 72 h intervals. Treated mice had reduced body weight loss, absence of stress (piloerection and hypotrichosis) and renal or hepatic abnormalities compared to the AmB deoxycholate treated group. In addition, the formulation raised no genotoxic and cytotoxic effects (Amaral et al., 2009). A subsequent study showed that the nano-D-AMB is highly captured in the lungs, liver, and spleen of mice (Souza et al., 2015). DMSA-PLGA NPs loaded with ITZ have also been studied against *P. brasiliensis* and the PLGA-ITZ had lower MICs compared to ITZ alone with less cytotoxicity compared to the free drug (Cunha-Azevedo et al., 2011).

Gallic acid is a secondary metabolite derived from plants such as *Paeonia rockii, Astronium* sp., and *Syzygium cumini*, among others. Interestingly, reversed gallic acid, or dodecyl gallate (DOD) has antifungal activity (Singulani et al., 2018). A recent study evaluated the antifungal efficacy of DOD associated with NLS (DOD + NLS) *in vitro* and *in vivo*. The results showed that the formulation exhibited good *in vitro* activity against *P. brasiliensis* and *P. lutzii* (0.24 and 0.49 mg/L, respectively), low toxicity in pulmonary fibroblasts (>250 mg/L) and zebrafish embryos (>125 mg/L). In addition, DOD + NLS reduced the fungal load in mouse lungs at a concentration of 10 mg/kg (Singulani et al., 2018).

Although promising, vaccines that have been studied against PCM have shown a rapid degradation of the immunogen (Travassos and Taborda, 2012). The most efficient way to protect the immunogenic molecule, reduce its concentration and reduce the number of doses was achieved by complexing it into NPs, as shown by Amaral et al. (2010), Jannuzzi et al. (2018), and Ribeiro et al. (2013). For example, the immunomodulatory peptide P10 trapped in PLGA NPs was effective in treating chronic murine PCM after 90 days of treatment with 5 or 10 50 μL^−1^, and the P10-PLGA NPs induced a robust, protective Th1 immune response (Amaral et al., 2010).

Variable single-chain fragments (scFv) obtained from the monoclonal antibody (mAb) 7.B12 that mimics gp43, the main *P. brasiliensis* antigen was incorporated into PLGA. After scFv-PLGA treatment of infected mice, a reduction in fungal load and increased production of IFN-γ and IL-12 cytokines was observed, as well as an abundance of macrophages and dendritic cells was seen in the lung tissue (Jannuzzi et al., 2018).

Ribeiro et al. (2013) used liposomes and PLGA to deliver plasmids containing the genetic information necessary for the expression of *Mycobacterium leprae* heat shock proteins (DNAhsp65). Both formulations were able to promote immune response modulation and fungal load reduction with the advantage of nasal administration of the liposomal formulation that could be more easily accepted by patients.

The studies involving NPs for PCM treatment are summarized in Table 3 and for vaccination in Table 4.

**Table 3:** Nanoformulations studied for the treatment of fungal infections caused by dimorphic fungi *Coccidiodes* sp., *Paracoccidiodes* sp. and *Histoplasma* spp..

**Nanoparticle**	Drug (^*^)	**Fungi**	***In vitro/in vivo***	**References**
Lipid complex; colloidal dispersion and liposomal	AmB and NYS	*C. immitis*	*In vitro*	González et al., 2002
Liposomal (AmBisome)	AmB	*C. immitis*	*In vivo* (human)	Antony et al., 2003; Rangel et al., 2010; Nakhla, 2018; Sidhu et al., 2018
			*In vivo* (rabbits)	Clemons et al., 2002
		*C. posadasii*	*In vivo* (rabbits)	Clemons et al., 2009
		*Coccidiodes* spp.	*In vivo* (human)	Stewart et al., 2018
		*H. capsulatum*	*In vivo* (human)	Johnson et al., 2002
Lipid complex (Abelcet)	AmB	*C. immitis*	*In vivo* (human)	Koehler et al., 1998; Sidhu et al., 2018
		*C. posadasii*	*In vivo* (rabbits)	Capilla et al., 2007; Clemons et al., 2009
PLGA-DMSA	AmB	*P. brasiliensis*	*In vivo* (mice)	Amaral et al., 2009; Souza et al., 2015
	ITZ		*In vitro*	Cunha-Azevedo et al., 2011
Nanostructured lipid system (NLS)	Dodecyl gallate (DOD)	*P. brasiliensis* and *P. lutzii*	*In vitro/in vivo*	Singulani et al., 2018

(*) * Drugs: AmB, Amphotericin B; ITZ, Itraconazole; NYS, Nystatin*.

**Table 4:** Studies reporting the use of nanoformulations as an antigen delivery vehicle for *Candida albicans* and *Paracoccidioides brasiliensis* vaccine.

**Fungi**	**Nanoformulation + adjuvant**	**Antigen**	**Animal model**	**Route of administration**	**References**
*Candida albicans* and *C. tropicalis*	PC/Chol liposomes	Fraction of *C. albicans* mannan	BALB/cByJ mice	Intravenous	Han and Cutler, 1995
*Candida albicans*	DMPC/DMPGLiposomes + LA	Ribosomes of *C. albicans*	ICR mice	Subcutaneous	Eckstein et al., 1997
	Metallochelating liposomes + MDP	rHSP90	BALB/c mice	Intradermal	Mašek et al., 2011
	Metallochelating liposomes + MDP and GMDP	rHSP90	ICR mice	Intradermal	Knotigová et al., 2015
	MO liposomes + DODAB	*C. albicans* wall proteins	BALB/c mice	Subcutaneous	Carneiro et al., 2015, 2016
*Paracoccidiodes brasiliensis*	PLGA	P10 peptide	BALB/c mice	–	Amaral et al., 2010
		Single-chain Variable fragments (scFv)	BALB/c mice	Intramuscular	Jannuzzi et al., 2018
	PLGA e Liposomes	DNAhsp65	BALB/c mice	Intramuscular or intranasally	Ribeiro et al., 2013

### Histoplasmosis

Histoplasmosis is an invasive endemic mycosis caused by the thermo dimorphic fungus *H. capsulatum* (Kauffman, 2007). Histoplasmosis is considered the most common respiratory fungal infection with a worldwide distribution of an asymptomatic infection to deep pulmonary mycosis and/or systemic disease, depending on the infectious inoculum and the immunologic condition of the host (Sepúlveda et al., 2017). The yeast form of *H. capsulatum* has mechanisms to prevent intracellular death by phagocytes. In addition, the intracellular localization of the fungus makes it difficult to treat the infection, as macrophages can act as a barrier, preventing the antifungal drug from interacting with its target in the cell (Edwards et al., 2013).

Currently, only AmBisome® has been evaluated in the treatment of histoplasmosis. A study compared the efficacy of AmB deoxycholate and AmBisome® in the treatment of 81 patients with moderate and severe histoplasmosis associated with AIDS. The AmB deoxycholate achieved clinical success in 14 of 22 patients (64%), with death of 3. Side effects developed in 63%, with nephrotoxicity in 37%. AmBisome® was effective in 45 of 51 patients (88%), although one patient died, 25% experienced drug-related side effects with 9% developing nephrotoxicity (Johnson et al., 2002) (Table 3).

Although the design of nanoparticles for delivery to specific cells is critical (as mentioned in topic 1), we believe that it is only a matter of time before these strategies are more deeply explored and improved upon in relation to fungal infections caused by pathogens such as *H. capsulatum*. These strategies can enhance the efficacy of treatment and, potentially, reduce occurrences of the disease.

There is no NP-based strategy for vaccination against *H. capsulatum*, although there are studies based on glucan particles extracted from *S. cerevisiae* (Wüthrich et al., 2015; Deepe et al., 2018).

### Coccidioidomycosis

Coccidioidomycosis is caused by inhalation of infectious propagules of the dimorphic fungus *C. immitis* or *C. posadasii*. It is an endemic mycosis in the southwestern USA, Mexico, and some regions of South America (Stockamp and Thompson, 2016). Studies indicated that 17–29% cases of pneumonia acquired in highly endemic areas were caused by *Coccidioides* spp. (Thompson, 2011). Patients may have varying complications of the disease, from pulmonary to widespread infections reaching bones, joints, meninges, and skin (Thompson, 2011; McConnell et al., 2017).

Prior to nanoformulations, it was believed that the use of AmB to treat meningitis was not possible due to the low concentration of drugs reaching the brain. However, AmBisome®, was effective in animal models (Clemons et al., 2002). The increased ability of liposomal AmB to reach the disease site is due to the transport by infiltrating monocytic cells (Clemons et al., 2009). The authors compared the efficacy of liposomal AmB formulation of Abelcet® and AmBisome® in the treatment of meningitis caused by *C. posadasii* in New Zealand white rabbits. The treated animals showed a reduction in fungal burden on the brain and spinal cord, 100–10,000 times lower than the untreated group. Statistically, both formulations were considered equally efficient. However, as we described, a formulation with higher liposomal effects has been described for drug delivery to the brain for the treatment of cryptococcoccal meningitis (Xu et al., 2011).

To evaluate the efficacy and toxicity of Abelcet® and AmBisome® in the treatment of severe coccidioidomycosis in human patients, a retrospective review was conducted in patients between 2005 and 2014. Both formulations were equally effective in the treatment without significant difference, however, due to acute kidney injury, the treatment had to be discontinued in 10 patients treated with Abelcet® and in only one with AmBisome® (Sidhu et al., 2018). The toxicity of the Abelcet® has previously been discussed (Koehler et al., 1998).

AmBisome® is effective in treating coccidioidomycosis in human patients (Stewart et al., 2018). During treatment of coccidioidal meningitis for a period of 9 months, AmBisome® did not present clinical or laboratory data suggestive of toxicity (Rangel et al., 2010). In addition, AmBisome® has been successful in treating disseminated coccidioidomycosis in patients undergoing steroid therapy (Antony et al., 2003). It was employed in case of rare dissemination to the spine that required surgical intervention, being associated with continuous therapy with other azoles (Nakhla, 2018).

The studies involving NPs in the treatment of coccidioidomycosis are summarized in Table 3.

*Blastomyces* sp. and *Pneumocystis* sp. are important pathogens that cause systemic infections. The effectiveness of nanoformulations against these genera has not yet been discussed in the literature.

## Concluding Remarks

The search for new and more effective therapeutic options for the treatment of fungal infections has advanced continuously with the use of new technologies such as the development of NPs. Different types of nanoformulations have been studied and greater efficacy and less toxicity have been achieved in the administration of conventional antifungal drugs, such as AmB, compared to the free drug available in today's market. In this way, nanotechnology allows for the development of formulations that can improve not only the effectiveness of the treatment, but also the quality of life of the patient by reducing side effects, especially during prolonged therapies. In addition, we emphasize here the importance of developing new drugs that can overcome resistance and that can be combined with NPs in the development of improved therapies. Nanotechnology is still an expanding field in vaccinology and pharmacology. The application of NPs for antigen delivery is at an early stage of development, but the first studies already show the advantages of this system, as described in this review. In addition, NPs can be obtained by different synthetic methods that allow for the adaptation of production according to the needs of the manufacturer. Obstacles, however, such as the standardization of NPs still need to make progress in this field.

## Author Contributions

BK conceived, designed, did the literature review, provided, and wrote the manuscript. SR and SS did the literature review, provided, and wrote the manuscript. JN, LT, and CT assisted in the preparation, final review, and co-wrote the manuscript. All authors listed approved it for publication.

## Conflict of Interest

The authors declare that the research was conducted in the absence of any commercial or financial relationships that could be construed as a potential conflict of interest.

## Abbreviations

AmB: Amphotericin B
ITZ: Itraconazole
NPs: nanoparticles
PLGA: Poly (lactic-co-glycolic acid)
PEG: polyethylene glycol
NLCs: Nanostructured lipid carriers
SLNs: solid lipid Nanoparticles
PAMAM: poly (amidoamine)
AgNPs: Silver nanoparticles
AuNPs: gold nanoparticles.
